# Endoglin (CD105) and proliferation index in recurrent glioblastoma treated with anti-angiogenic therapy

**DOI:** 10.3389/fonc.2022.910196

**Published:** 2022-09-06

**Authors:** António G. P. Bastos, Bruno Carvalho, Roberto Silva, Dina Leitão, Paulo Linhares, Rui Vaz, Jorge Lima

**Affiliations:** ^1^ Faculty of Medicine of the University of Porto, Porto, Portugal; ^2^ Department of Neurosurgery, Centro Hospitalar Universitário S. João, Porto, Portugal; ^3^ Institute for Research and Innovation in Health (i3S), R. Alfredo Allen Porto, Porto, Portugal; ^4^ Department of Pathology, Centro Hospitalar Universitário S. João, Porto, Portugal; ^5^ Neurosciences Center-CUF Hospital, Porto, Portugal; ^6^ Institute of Molecular Pathology and Immunology, University of Porto (Ipatimup), Porto, Portugal

**Keywords:** glioblastoma, CD105, endoglin, Ki-67, glomerular vascular proliferations, bevacizumab, overall survival, progression-free survival

## Abstract

**Introduction:**

CD105 is an angiogenic biomarker that is useful to determine the microvessel density (MVD) within a tumor, namely, in highly vascularized tumors like glioblastoma (GBM). However, its expression has shown inconsistent associations with the prognosis of GBM patients. The aim of this study was to evaluate the value of MVD-CD105 (microvessel density assessed with anti-CD105 antibody) and Ki-67 (proliferation index marker) as prognostic and therapy response biomarkers, specifically in primary tumors and in recurrent tumoral specimens of a cohort of GBM patients treated with bevacizumab upon recurrence.

**Materials and methods:**

We conducted a retrospective study of 102 consecutive GBM patients treated with bevacizumab upon recurrence at CHUSJ between 2010 and 2017. Demographic, clinical, and survival data of all patients were collected and analyzed. The tissue expression of MVD-CD105 and Ki-67 in primary and recurrent specimens was correlated with progression-free survival after temozolomide (PFS-1), progression-free survival after bevacizumab (PFS-2), and overall survival (OS).

**Results:**

The immunohistochemical expression score for MVD-CD105 was similar in primary and recurrent tumoral specimens (mean scores of 15 and 16, respectively). Likewise, the mean Ki-67 expression was similar in primary (mean of 31% of tumor cells) and recurrent tumoral specimens (mean of 29% of tumor cells). MVD-CD105 expression in primary tumors had no impact on PFS-1, PFS-2, or OS. At recurrence, patients whose tumors showed increased MVD-CD105 had worse median PFS-2 (2 vs. 8 months, *p* = 0.045) and OS (17 vs. 26 months, *p* = 0.007) compared to those whose tumors showed lower MVD-CD105. CD105 tumoral pattern and localization had no impact on prognosis. Ki-67 expression was not associated with differences in survival outcomes.

**Conclusion:**

In this study, higher MVD-CD105 expression in recurrent GBM patients seems to be associated with a worse PFS-2 and OS while portending no prognostic significance in the primary tumors. This highlights the importance of keeping track of the molecular evolution of the tumor over the course of the disease.

## Introduction

Glioblastoma (GBM) is the most common primary malignant brain tumor in adults accounting for 48.3% of all malignant tumors of the central nervous system (CNS). Although some considerable efforts in the development of new treatment approaches have been made, the prognosis remains ominous, even with the best surgical resection, radiotherapy, and chemotherapy. In fact, the median overall survival (OS) has not increased significantly and the 5-year survival rates are around 5% to 6% (1). It is widely acknowledged that the incurable nature of GBM is primarily attributable to its infiltrative growth and that is why primary treatment is followed by recurrence in virtually all patients (2).

Many efforts have been made to find appropriate treatments for GBM recurrence after primary therapy. While surgical resection of recurrent GBM seems effective in a minority of patients, multiple systemic therapies have been more commonly used. In particular, angiogenic inhibitors appeared as promising agents due to the high vascularization and angiogenic activity of this tumor (3). The most used anti-angiogenic drug in GBM is an anti-VEGF monoclonal antibody, bevacizumab (BEV). Although this treatment showed efficacy, with nearly 50% responders, its effect was transient due to secondary anti-angiogenic resistance (4). In the past years, several tumor markers have been investigated for their role in the prognosis and therapeutic response of GBM patients (5).

CD105 (also known as endoglin), a receptor for transforming growth factors 1 and 3 (TGF-β1 and TGF-β3), plays a key role in angiogenesis and vasculogenesis during tumoral development (6). CD105’s expression is upregulated on actively proliferating endothelial cells, mainly in immature vessels. It also promotes new vascular networks, which potentiates further glioma development through oxygen supply, thus enhancing tumoral invasion (7, 8). Some studies suggest that CD105 preferentially marks novel angiogenic vessels, which means that it is a sensitive and specific biomarker of angiogenesis within the tumor, with a specificity of 72% and a sensitivity of 80% (7, 9). While some data have shown a correlation between an elevated microvessel density (MVD), assessed with anti-CD105 (MVD-CD105), and a worse survival/prognosis of GBM patients (10–12), others have shown no specific association (5, 13). However, to our knowledge, there are no studies that additionally evaluated the correlation between MVD-CD105 and the prognosis of recurrent GBM patients treated with BEV. A recent meta-analysis studying the effect of this drug in the treatment of newly diagnosed GBM has concluded that BEV is associated with prolonged progression-free survival (PFS), but there is an inconsistent effect on the OS of unselected patients; therefore, there is a need to identify subpopulations of patients who may benefit from this therapy (14).

Ki-67 antibody is an IgG class monoclonal antibody that is useful to distinguish between proliferating and non-proliferating cells. Furthermore, the percentage of proliferating cells (Ki-67 labeling index) can be used to discriminate more aggressive tumor phenotypes (15). The results of a meta-analysis that included 51 studies covering 4,307 patients showed that Ki-67 positivity was significantly correlated with poor OS and PFS in patients with glioma (16). Despite this, little is known about the impact of the cell proliferation index on the outcome of patients with recurrent GBM treated with anti-angiogenics.

The aim of this study was to evaluate the value of MVD-CD105 and Ki-67 as prognostic and therapy response biomarkers in primary tumors and in recurrent GBM treated with bevacizumab upon recurrence.

## Materials and methods

### Patient and tissue collection

We conducted a retrospective study of 102 consecutive patients with recurrent GBM, diagnosed and treated at Centro Hospitalar Universitário S. João (CHUSJ) between 2010 and 2017. All patients were submitted to surgery and treated with the Stupp protocol [standard first-line chemotherapy with temozolomide (TMZ), administered 75 mg/m^2^ concurrent with daily external-beam radiation therapy (RT) (2 Gy/fraction, for a total of 60 Gy in 30 fractions), followed by adjuvant TMZ at 150–200 mg/m^2^ for 5 days every 28 days until progression]. At recurrence, all patients were treated with BEV-based therapy, namely, BEV (10 mg/kg) in monotherapy or plus irinotecan (340 or 125 mg/m^2^, with or without antiepileptic drugs, respectively) or lomustine (90 mg/m^2^). We also collected 19 recurrence specimens obtained *via* a second or third resection surgery.

The inclusion criteria were as follows: i) patients with histologically proven GBM and age ≥18 years; ii) first-line therapy according to the Stupp protocol; iii) recurrence assessment according to the response assessment in neuro-oncology (RANO) criteria; and iv) second-line treatment with BEV-based therapy after multidisciplinary neuro-oncology team meeting decision. We included all patients with representative tumoral specimens, operated between 2010 and the end of 2017, who matched the eligibility criteria and followed them until death or last follow-up.

The diagnosis of GBM was centrally reviewed by a neuropathologist (RS). This was followed by immunohistochemical (IHC) analysis of Ki-67 and CD105, which was performed in representative tumoral sections.

Demographic, clinical, therapeutic, and survival data of all patients were collected and analyzed through the CHUSJ electronic clinical registries. This included the analysis for potential confounders, mainly age, extent of resection, focality, and Eastern Cooperative Oncology Group (ECOG) status. The tissue expression of Ki-67 and CD105 in primary and recurrent specimens was correlated with survival data, more specifically, OS, PFS after TMZ (PFS-1) defined as the time from initiation of TMZ until progression, and PFS after BEV (PFS-2), meaning time until recurrence after introducing BEV-based therapy.

### Immunohistochemistry study

Immunohistochemistry was performed using an automated Ventana BenchMark ULTRA Staining System, using the OptiView DAB IHC Detection Kit (Roche/Ventana Medical Systems, Tucson, AZ, USA) according to the manufacturer’s instructions. Tissue sections were obtained from a selected paraffin-embedded block, which was serially cut with a microtome (Microm HM 325, Thermo Scientific™, Braunschweig, Germany) at 2–3 µm thickness and placed on positively charged microscopic slides (Superfrost Plus™ from Thermo Scientific™, Braunschweig, Germany). Heat-induced epitope retrieval was carried out using Cell Conditioning 1 (CC1) solution, followed by blocking endogenous peroxidase with 3% hydrogen peroxide solution.

Primary antibodies with monoclonal antibodies for Ki-67 (RTU, MIB-1, Roche/Ventana Medical Systems, Tucson, AZ, USA) and CD105 (endoglin, 1/100, SN6h, BioLegend, San Diego, USA) were used according to the manufacturer’s instructions and were added manually using the Ventana™ BenchMark ULTRA equipment and the OptiView™ Universal DAB detection kit (Ventana Medical Systems, AZ, USA).

The positive controls, tissue components to confirm that the antibody applied functioned properly, were used in all the slides. A negative control slide was used, in place of the primary antibody to evaluate non-specific staining, using a specific reagent, Rabbit Monoclonal Negative Control Ig (Ventana Medical Systems, AZ, USA, 790-4795). After IHC staining, slide washing was performed with EZPrep (2 × 10 min) and running water (2× until clean), followed by dehydration with ethanol 100% (2 × 10 min) and, finally, diaphanization with xylene (2 × 10 min). The slides were mounted with a permanent mounting medium (Histofluid, Paul Marienfeld GmbH & Co. KG, Lauda-Kὃnigshofen, Deutschland) for optical microscopy analysis.

### Scoring and interpretation of immunochemistry

Sections were examined for CD105 immunoreactivity by a neuropathologist blinded to the outcomes and clinical features. The vascular hotspot method was used, which involves identifying areas of higher MVD within the tumor. These areas were found by scanning tumoral sections at low power (×10–×100). From each tumor specimen, three hotspots were chosen. Within each hotspot, vessel counting was performed in a high-power field (×200/0.1 mm^2^). Any stained endothelial cell clearly separated from the adjacent tissue was regarded as a separate vessel. The number of CD105-positive vessels/0.1 mm^2^ of tumor tissue was calculated in three hotspots and its average was used to indicate MVD (17–19).

Furthermore, the Ki-67 index of each specimen was determined by a neuropathologist and was defined as the percentage of immunoreactive tumoral cell nuclei among the total number of cells (15).

Information regarding the tumoral pattern (non-specific, diffuse, or glomeruloid) and location [peritumoral brain zone (PBZ), non-specific, or tumoral core (TC)] of CD105 expression was also determined.

### Data and statistical analysis

The primary endpoint of this study was to correlate the expression of CD105 and Ki-67 with survival data. MVD-CD105 and Ki-67 expression levels were dichotomized above and below the mean expression. Differences between groups were analyzed using the Mann–Whitney *U* test, Fisher’s exact test, and chi-square test, as appropriate. Correlation between continuous variables was analyzed *via* Pearson’s correlation coefficient. Survival data were evaluated using the Kaplan–Meier product-limit analysis and 95% confidence intervals (CI) were calculated. The log-rank test was used to detect statistically significant differences in survival distributions. Potential confounders and effect modifiers were assessed, and the impact on the outcomes was evaluated through the log-rank test. Differences were considered statistically significant at *p <*0.05. The software used for the statistical analysis was IBM SPSS Statistics 27.

### Ethical approval

This study was approved by the Local Ethical Committee of Centro Hospitalar Universitário S. Joaão (Porto, Portugal) (no. 17/21) and conducted according to the National Ethical Guidelines.

## Results

### Patients’ demographics

Among the 102 GBM patients, there were 36 women (35.3%) and 66 men (64.7%). The median age at diagnosis was 58 years, ranging from 26 to 77. Most of the patients (90.2%, *n* = 92) had an ECOG status of 0 or 1, while 10 patients (9.8%) had an ECOG status of 2–3. The median OS was 19 months, while PFS-1 and PFS-2 were 8 and 5 months, respectively. Regarding the extent of resection, total resection was achieved in 52 (51%) patients, partial resection in 39 (38.2%) patients, and biopsy in 8 (7.8%) patients. Median follow-up time, defined as the median observation time for those patients who were event-free at the end of follow-up, was 26 months.

Seventeen patients were reoperated: 15 had one reoperation and 2 patients had two reoperations. The median age of the reoperated patients was 51 years. Regarding the first reoperation, 15 patients (88.2%) had a presurgical ECOG performance status of 0–1, while total resection was achieved in 10 patients (58.8%).

Full demographic and clinical data are depicted in **Tables 1**, **2**.

**Table 1:** Demographic and clinical parameters of the cohort.

Demographic or clinical parameter	*N* (%)	Median (range)	Median (95% CI)
**Gender**			
Female	36 (35.3%)		
Male	66 (64.7%)		
**Age**		58 years (26–77)	
**ECOG status**			
0	33 (32.4%)		
1	59 (57.8%)		
2	8 (7.8%)		
3	2 (2.0%)		
**Focal vs**. m**ultifocal**			
Focal	89 (87.3%)		
Multifocal	10 (9.8%)		
Missing data	3 (2.9%)		
**Type of resection**			
Total resection	52 (51%)		
Partial resection	39 (38.2%)		
Biopsy	8 (7.8%)		
Missing data	3 (3%)		
**Number of TMZ cycles**		6 (1–42)	
**Cumulative dosage of BEV**		90 mg/kg (10–640)	
**PFS-1**			8.00 months (6.77–9.23)
**PFS-2**			5.00 months (3.72–6.29)
**OS**			19.00 months (16.67–21.33)

**Table 2:** Demographic and clinical parameters of patients submitted to reoperation.

Demographic or clinical parameter	*N* (%)	Median (range)	Median (95% CI)
**Age**		51 years (30–64)	
**Gender**			
Female	7 (41.2%)		
Male	10 (58.8%)		
**ECOG status**			
0	6 (35.3%)		
1	9 (52.9%)		
2	0 (0%)		
3	2 (11.8%)		
**Extent of resection**			
Total resection	10 (62.5%)		
Partial resection	6 (37.5%)		
Biopsy	0 (0%)		
Missing data	1 (1%)		
**PFS-1**			11.00 months (7.77–14.23)
**PFS-2**			6.00 months (0.12–11.88)
**OS**			21.00 months (15.35–26.65)

### Survival data

**Figure 1:**
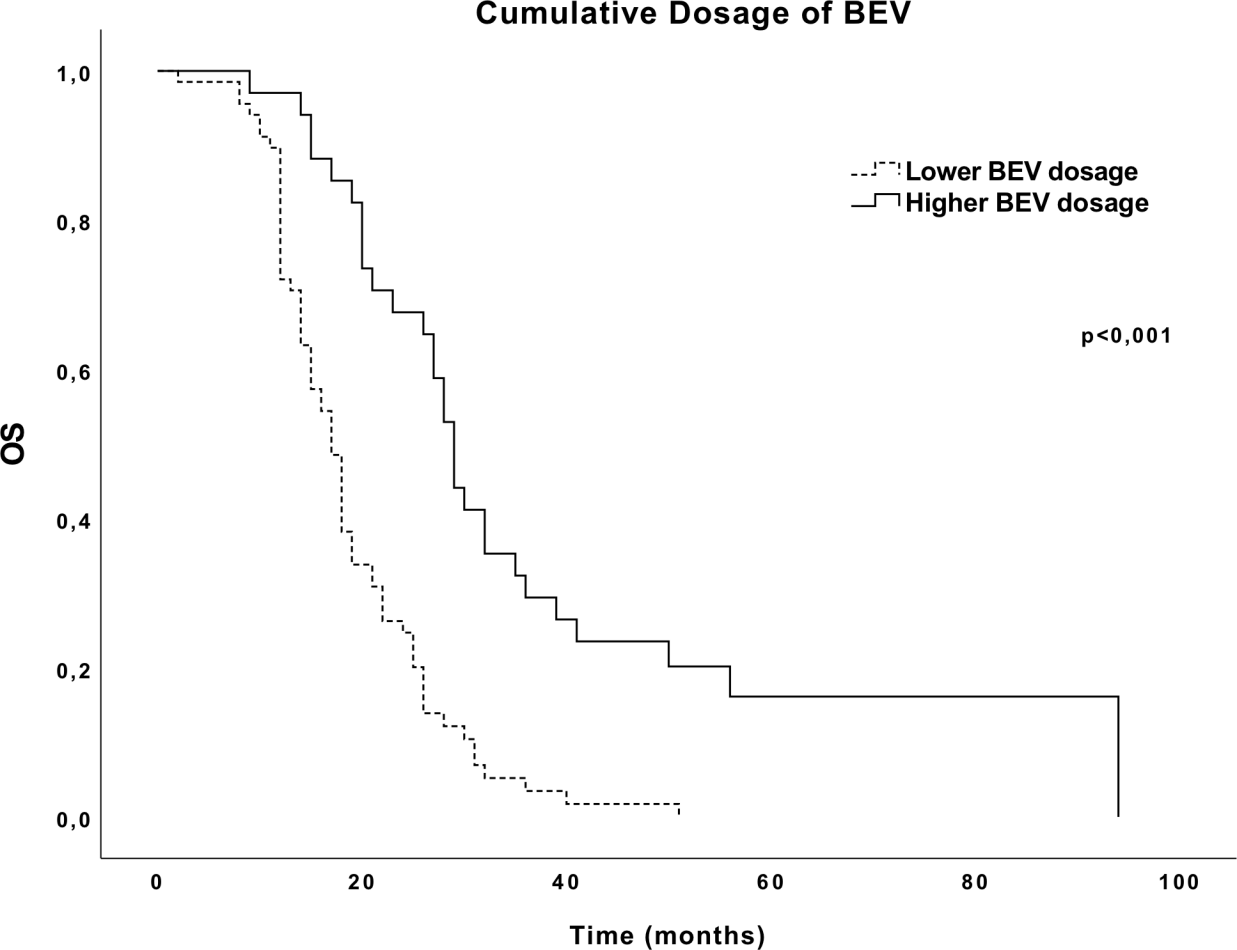
Overall survival (OS) Kaplan–Meier curves according to the cumulative dosage of BEV.

The median OS of all cohorts was 19 months (95% CI 16.67–21.33), the median PFS during TMZ (PFS-1) was 8 months (95% CI 6.77–9.23), and the median PFS while on BEV (PFS-2) was 5 months (95% CI 3.72–6.29). Reoperated patients had a median OS of 21 months (95% CI 15.35–26.65), although not statistically different from patients submitted to a single surgery. Patients who received a higher cumulative dosage of BEV were associated with a better OS, in comparison to those who received a lower cumulative dosage of BEV (29 vs. 17 months, *p* < 0.001, respectively) (**Figure 1**).

### MVD-CD105 and Ki-67 expression

**Figure 2:**
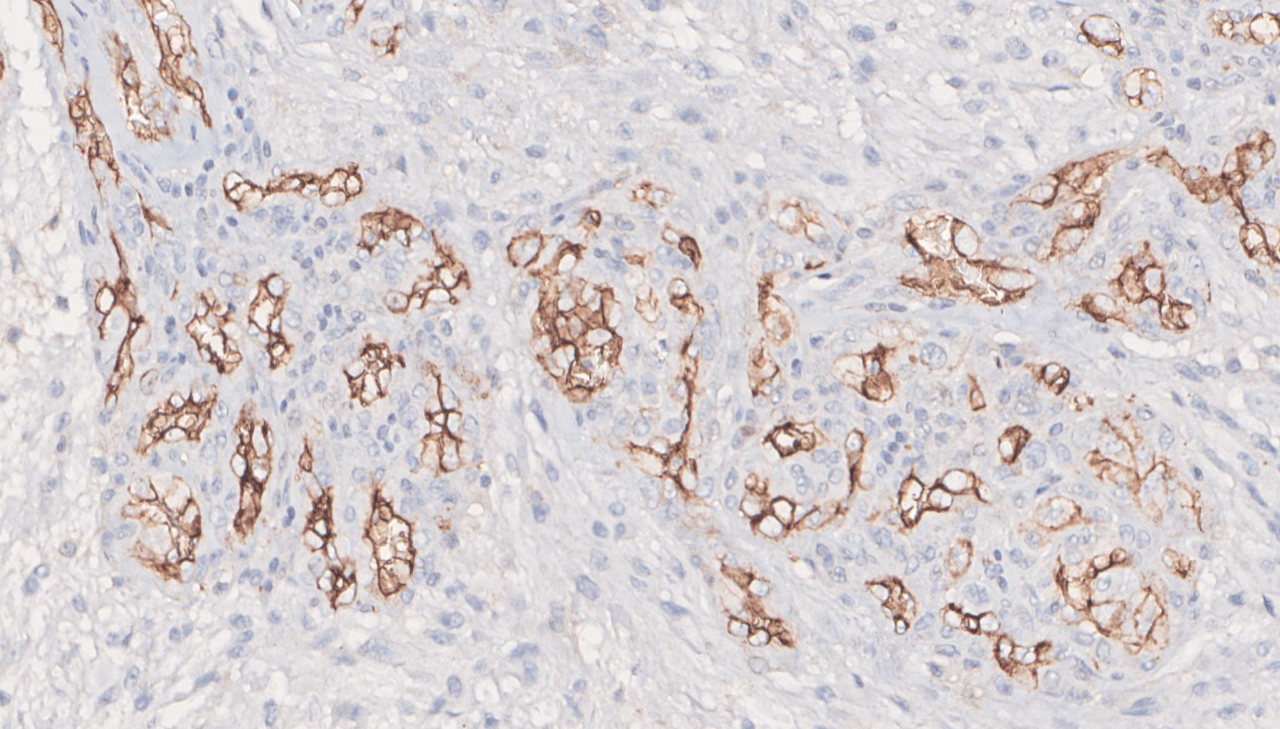
Representative positive immunohistochemical staining with CD105 glomeruloid pattern expression (original magnification ×200).

Among the primary tumors, 94.8% (*n* = 91) showed CD105 positivity, while 5.2% (*n* = 5) had no CD105 expression. The mean MVD-CD105 expression in the 96 primary specimens analyzed was 15, ranging from 0 to 42.7 (SD: 9.84). Furthermore, in the recurrent tumoral specimens available (*n* = 16), 94% (*n* = 15) showed CD105 positivity, while 6% (*n* = 1) had no CD105 expression; a similar mean MVD-CD105 expression level was observed (16; SD: 12.56). Representative images of CD105 immunopositivity are depicted in **Figure 2**.

Regarding Ki-67 expression, in the 80 primary specimens analyzed for this biomarker, the mean was 31%, ranging from 5% to 90% (SD: 17.3%). In the recurrent tumor specimens available, the mean Ki-67 expression was 29% (SD: 10.3%).

MVD-CD105, Ki-67, tumoral pattern, and location of CD105 expression data from primary and recurrent specimens are fully described in **Table 3**.

**Table 3:** MVD-CD105, Ki-67, tumoral pattern, and location of CD105 expression.

	*N* (%)	Mean (SD)
**MVD-CD105**		
Primary tumor -Positivity	96 91 (94.8%)	15.42 (9.84)
Recurrent tumor -Positivity	16 15 (94%)	16.49 (12.56)
**Ki-67**		
Primary tumor	80	31% (17.32%)
Recurrent tumor	5	29% (10.30%)
**Tumoral location (CD105 expression)**		
Primary tumor -Tumoral core -Peritumoral brain zone -No staining -No specific location	96 81 (84.5%) 8 (8.3%) 5 (5.2%) 2 (2.1%)	
Recurrent tumor -Tumoral core -Peritumoral brain zone -No staining	16 12 (75%) 3 (18.8%) 1 (6.3%)	
**Tumoral pattern (CD105 expression)**		
Primary tumor -No specific pattern -Glomeruloid pattern -No staining -Diffuse pattern	96 72 (75%) 17 (17.7%) 5 (5.2%) 2 (2.1%)	
Recurrent tumor -No specific pattern -Glomeruloid pattern -No staining -Diffuse pattern	16 13 (81.3%) 2 (12.5%) 1 (6.3%) 0(0%)	

**Figure 3:**
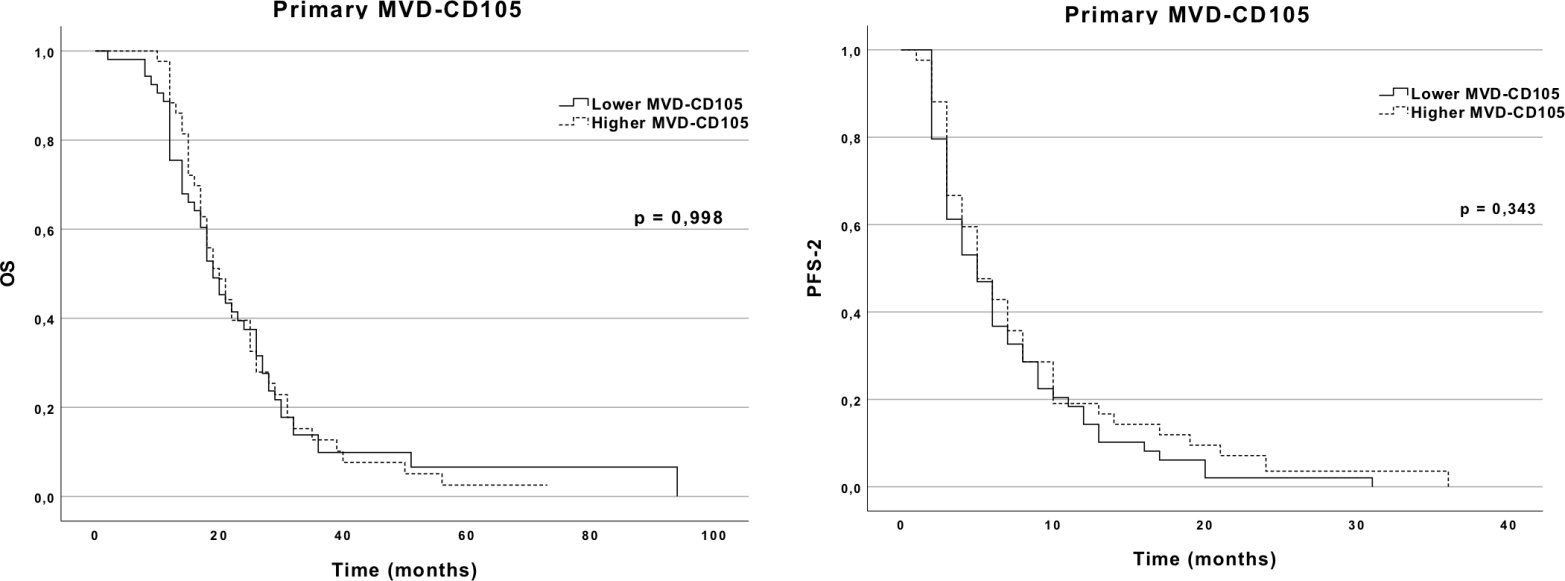
OS and PFS-2 Kaplan–Meier curves according to MVD-CD105 expression in the primary GBM.

In primary tumors, we found no statistically significant difference in the outcomes of patients with higher (above mean) and lower expression (below mean) of CD105 regarding PFS-1 (9 vs. 8 months, *p* = 0.388, respectively), PFS-2 (5 vs. 5 months, *p* = 0.343, respectively), and OS (20 vs. 19 months, *p* = 0.998, respectively) (**Figure 3**). Likewise, the mitotic index (Ki-67) level (above vs. below mean) was not statistically correlated with a better or worse OS (20 vs. 21 months, *p* = 0.619, respectively), PFS-1 (7 vs. 9 months, *p* = 0.569, respectively), or PFS-2 (5 vs. 6 months, *p* = 0.675, respectively). Additionally, we found no significant correlation between Ki-67 and MVD-CD105 expression (Pearson’s *r* = 0.05; *p* = 0.420). There were no differences between the different types of CD105 expression patterns and expression location concerning PFS-1, PFS-2, or OS.

**Figure 4:**
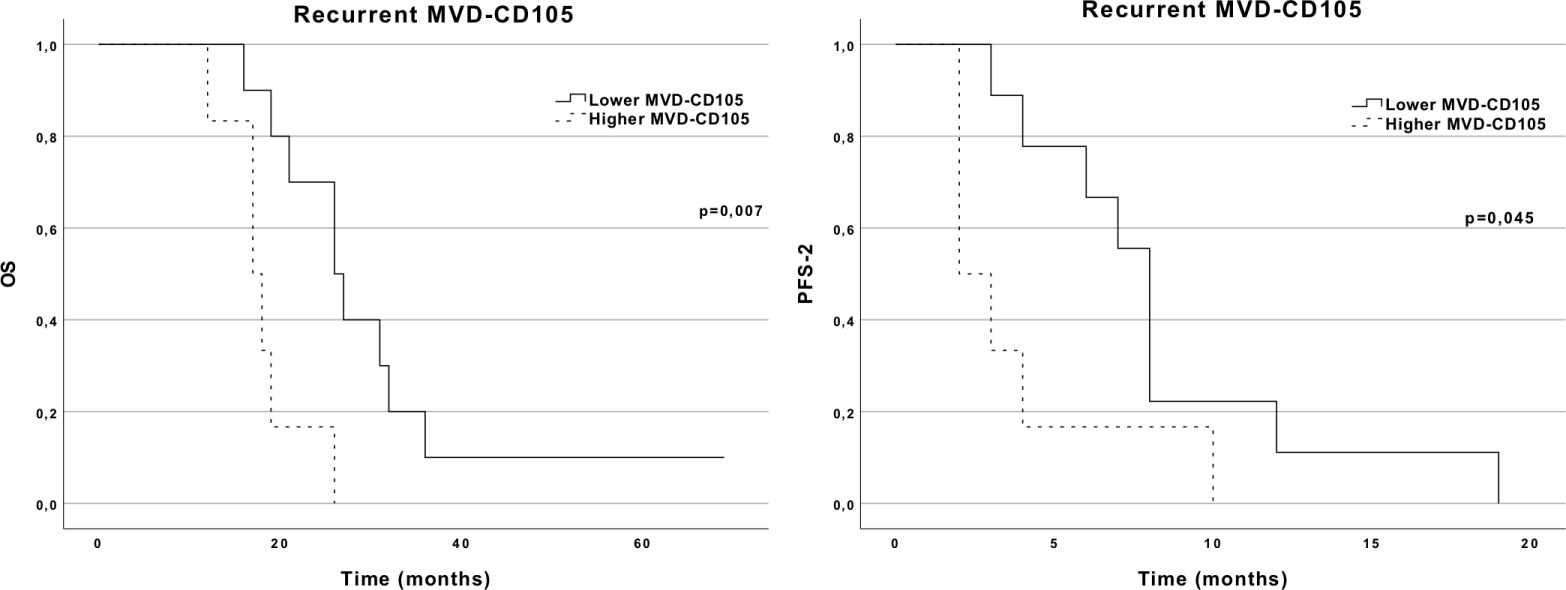
OS and PFS-2 Kaplan–Meier curves according to MVD-CD105 expression in the recurrent tumor.

Although we did not find differences in the primary tumor analysis, the same was not observed in the recurrent specimens. When analyzing recurrent GBM, increased MVD-CD105 was associated with an OS of 17 months, while low MVD-CD105 was associated with an OS of 26 months. The differences between these two groups were statistically significant (*p* = 0.007). PFS-2 was also significantly shorter in the group with a higher MVD-CD105 (2 months) in comparison to the lower MVD-CD105 group (8 months; *p* = 0.045) (**Figure 4**). A comparative analysis of the baseline characteristics between the two groups of MVD-CD105 expression showed no differences regarding age (*p* = 0.514, Mann–Whitney *U* test), ECOG performance status (*p* = 0.584, Mann–Whitney *U* test), and extent of resection (*p* = 1.00, Fisher’s exact test). Regarding Ki-67 expression in the recurrent GBM, there were no differences in PFS-2 or OS.

We analyzed whether the commonly known prognostic factors such as age, preoperative ECOG status, and extent of resection had an impact on OS and PFS-2. While age and extent of resection did not significantly affect OS and PFS-2, patients with a worse ECOG status (2 and 3) had a significantly worse OS in comparison to those with a better ECOG (0 and 1) (12 vs. 26 months, *p* = 0.039, respectively). The same trend was observed for PFS-2 (7 vs. 2 months), although these differences were not statistically significant (*p* = 0.148).

The variation of MVD-CD105 from the primary specimen to the first recurrence specimen did not show any impact on the outcomes. Patients with increasing MVD-CD105 had a median OS of 19 months (95% CI 12.60–25.40) versus 26 months (95% CI 11.39–40.61) in patients with a decrease in MVD-CD105 expression, although these differences had no statistical significance (*p* = 0.171, log-rank). No significant relationship was found between CD105 expression pattern and location and OS or PFS.

## Discussion

GBM is one of the most aggressive tumors in humankind, mainly because it displays high angiogenic levels and the highest grade of vascular proliferation (9). This finding suggested the potential benefit of anti-angiogenic therapies, such as BEV, a monoclonal anti-VEGF-A antibody, which targets VEGF, an endothelial proliferating factor. Although BEV has shown a positive impact on PFS, the results of randomized clinical trials (RCTs) failed to show a positive effect on OS (14). This might happen due to unclear resistance mechanisms (20). Additionally, alternative angiogenic pathways have been investigated as drug targets. Both glioma cells and glioma-associated endothelial cells produce TGF-β superfamily ligands that bind TGF-β receptors (TGF-βR). The TGF-βR type III endoglin (CD105) is a marker of proliferating endothelium that has already been studied as a potential therapeutic target (13).

CD105 is one of the most specific markers of newly formed endothelial cells, meaning it is more valuable in recognizing angiogenesis in neoplastic tissue in comparison to CD31 and CD34, which are pan-endothelial markers, staining also for normal endothelial cells. The expression levels of CD105 in the tumoral specimen seem to be more closely correlated with the expression of VEGF (in comparison to other endothelial biomarkers); thus, we can hypothesize that higher levels of CD105 in tumoral tissue would mean a better response to BEV as there would be more VEGF expressed within the tumor (9, 11).

In this study, we evaluated the prognostic and therapy response significance of CD105 and the proliferation index (Ki-67) in primary and recurrent tumoral specimens of a cohort of GBM patients. Of note, all recurrences were further treated with anti-angiogenic therapy.

When assessing primary tumoral specimens, higher or lower MVD-CD105 expression had no major impact on PFS-1, PFS-2, and OS. We identified five studies addressing MVD-CD105 and OS which reported variable prognostic effects of CD105 expression (5, 10–13). This might be due to different factors: the variability in the reactivity of endothelial cell antibodies, tissue pretreatment procedures, methodology for vessel counting used, and statistical analysis performed (11). Regarding the methodology used for IHC analysis, the majority used the same approach as in our study, while Burghardt et al. used a different IHC score for CD105 density, more specifically the histoscore (H-score) (13), and Polívka et al. used a quantitative approach (12). Studies that identified a correlation between an increase in MVD-CD105 and a worse OS were considered: Behrem et al. studied a smaller group of patients (46 patients), and other potential important variables that could influence the results were not analyzed (particularly preoperative ECOG status and extent of resection) (10); Yao et al. also found the same association as the previous study, but the group of patients studied was much different as it included all types of astrocytic tumors (11); Polívka et al. also found a statistical association but included a smaller group of patients (52 patients) (12). Our study did not find any significant association between primary MVD-CD105 expression and OS, which is in line with other studies, although we present a larger cohort of patients (*n* = 96) (5, 13).

Reviewing the current evidence, it is important to note that all studies on CD105 expression and its impact on OS and PFS did not limit their samples to patients exclusively treated with anti-angiogenics upon recurrence. This means that our study is unique as it only includes patients treated with BEV and we were able to assess the relationship between CD105 expression in available recurrent GBM and clinical outcomes. To our knowledge, no study has analyzed the association between MVD-CD105 and progression-free survival in patients submitted to anti-angiogenics (PFS-2). It could be anticipated that patients with higher MVD-CD105 expression would respond better to anti-angiogenic agents, since these are the ones with presumably higher VEGF levels—the therapeutic target of BEV. However, we observed the opposite, i.e., higher expression of CD105 in recurrent specimens was associated with more aggressive disease and lower progression-free survival while on BEV (PFS-2).

Herein, patients who received a higher cumulative dosage of BEV had a better OS (29 months) in comparison to those receiving a lower dosage (17 months). However, previous RCTs studying BEV in GBM have shown no positive impact of BEV on OS (14). This result might be explained by the fact that patients with a higher cumulative dosage of BEV are those who, in fact, better respond to this therapy and, consequently, have a longer duration of treatment.

Regarding Ki-67, although a recent meta-analysis pointed to a predictor utility of this proliferation biomarker, our study did not disclose any differences regarding prognostic outcomes. As the authors pointed out, the conclusion of the meta-analysis may be affected by publication biases, namely, fewer publications reporting the absence of prognostic associations (16). Furthermore, since higher levels of CD105 and Ki-67 seem to be associated with more aggressive tumors, we investigated whether these two markers were associated (10–12, 16). Although Behrem et al. found a statistically significant correlation between the two biomarkers in 46 patients with GBM, our study did not disclose any association (10).

We also analyzed tumoral location and pattern for CD105 expression in the GBM specimen. Tamura et al. showed that tumoral cells mainly located in the peripheral brain zone (PBZ) or infiltrative zone had a molecular composition suggesting the presence of more immature vessels, while tumoral cells located in the core had a positive expression for VEGF receptors 1 and 2, which meant a tumor with more mature blood vessels. The immature vessels in PBZ could be resistant to BEV therapy (21). We would predict that aggressiveness would be higher in tumors with PBZ CD105 infiltration in comparison to CD105 localized to the tumoral core. However, we did not find any association between TC and PBZ CD105 localization and PFS-2 and OS. Glomeruloid vascular proliferations (GVPs) are composed of multiple layers of endothelial cells with a high degree of proliferation, resulting in a more aggressive tumoral behavior and a worse prognosis in GBM patients. Furthermore, the formation of a larger GVP with tumoral necrosis and hypoxia could be an important cause of relapse to BEV therapy (22). Despite this, we did not find any prognostic difference between patients with a glomeruloid pattern in their tumoral specimens in comparison to other patterns.

To our knowledge, we show for the first time that higher MVD-CD105 at recurrence is correlated with a worse OS (17 vs. 26 months) and PFS-2 (2 vs. 8 months) in patients treated with anti-angiogenic. GBM is a very heterogeneous disease composed of a variety of subclonal populations with different genetic, transcriptomic, and functional characteristics. Inevitably, this leads to treatment resistance as some subclonal populations can escape to therapeutic agents. Consequently, when a tumor sample analyzed at recurrence is obtained, it will probably be genetically and phenotypically different from its respective primary tumor, highlighting the importance of capturing the molecular evolution of recurrent tumors, by analyzing both primary and recurrent samples (23). Only one study addressing CD105 expression has managed to compare newly diagnosed and recurrent GBM specimens from the same patient, showing no statistical difference in CD105 levels between the two (13). We confirmed that the average CD105 expression is similar in primary and respective recurrent samples, but also showed that a higher MVD-CD105 expression in the recurrent samples has a negative impact on OS (17 vs. 26 months) and PFS-2 (2 vs. 8 months) in patients further submitted to BEV-based therapy. This finding could be regarded as unexpected since a higher MVD-CD105 is associated with a higher VEGF expression and VEGF is the target of BEV therapy (11). The explanation could lie in the fact that higher MVD-CD105 means a higher degree of proliferation of the endothelial cells, which consequently enhance tumoral invasion and worsen the patient’s OS and response to BEV therapy (7, 8).

It has been previously shown that the inhibition of a specific factor can trigger feedback mechanisms that activate alternative oncogenic pathways (20). Zhang et al. demonstrated that when BEV is administrated (*in vitro* and *in vivo*), there is an inhibition of VEGF with an activation of the TGF-β1 pathway and a consequent upregulation of CD105 expression *via* the TGF-β–CD105–Smad pathway (24). There is also evidence from other cancers, such as colorectal cancer, that treatment with BEV therapy elevates the levels of CD105, in comparison to patients not treated with this drug. Therefore, CD105 could be an intrinsic or adaptive escape mechanism to anti-angiogenic therapy (BEV) (25). Interestingly, TRC105, a chimeric antibody targeting CD105, seems to enhance the effect of bevacizumab *in vivo* (26).

Studies targeting endoglin, as either monotherapy or combined with other (anti-angiogenic) therapies, have been performed. Although the clinical benefit was modest, the combined TRC105/anti-VEGF therapy appeared to be effective in VEGF therapy refractory patients and in preclinical models. Endoglin expression has also been reported in tumor-infiltrating Tregs, macrophages, cancer-associated fibroblasts, and cancer (stem) cells. This could contribute to the efficiency of TRC105, since targeting those cells might enhance antitumor responses. Additionally, preclinical studies have shown that immunomodulatory therapies increase TRC105 efficiency. In this perspective, the combination with checkpoint inhibitors might potentiate its efficiency. Considering the crosstalk between the endoglin and VEGF pathways, one might hypothesize that, in recurrent GBM patients highly expressing CD105, combined TRC105/anti-VEGF therapy could be a potential therapeutic strategy, reserving anti-angiogenic monotherapy for recurrent GBM patients with low levels of CD105 expression. It remains unknown whether these possible strategies benefit any subgroup of patients (27). Two phase II clinical trials have been performed to study the potential benefit of TRC105 and BEV in patients with recurrent GBM. The first one included patients previously treated with BEV who progressed and received TRC105 as an additional therapy. A median OS of 5.75 months which exceeded the 4.0 months seen in patients with BEV alone was observed (28). The second one studied the association of TRC105 with BEV in a population of BEV naive patients with recurrent GBM, compared with BEV-only-treated patients. Preliminary results did not show any statistical differences between the two arms regarding OS and PFS-2 (29).

We have shown in our study that CD105 expression in primary tumoral specimens had no impact on PFS or OS. The same was not true in the recurrent specimens, where a higher expression of CD105 was associated with a worst response to subsequent anti-angiogenic therapy. The fully described process of neoangiogenesis throughout GBM progression and its impact on the outcome at several timepoints of the disease is not known. Some authors have shown that temozolomide and bevacizumab can trigger different proliferative, apoptotic, and angiogenic responses. In fact, TMZ and BEV decrease GBM–endothelial cells and tube formation viability but only transiently. On the other hand, these agents promote a downregulation of p53 expression. Furthermore, although CD105 is highly expressed in activated GBM–endothelial cells in primary and recurrent tumors, its functional activity as an accessory protein of the transforming growth factor receptor might be different throughout the disease. This could justify the different correlations with the outcome at different points in the course of the disease. Serial analysis of gene expression of angiogenesis modulators could help to understand which collateral and different signaling pathways are driving aberrant new vessel formation and their relative instability (30). There is a shifting paradigm in which oncology clinicians and researchers should track the molecular evolution of the tumor with time, addressing different targets over the course of the disease. In this perspective, the availability of recurrent tumor samples is paramount. It can be of interest to study the administration of this combined therapy in GBM patients with higher levels of CD105 in the recurrent setting.

Some strengths and limitations of this study should be addressed. The strengths include a robust sample size, availability of recurrent specimens, IHC evaluation with central pathology review, and blinded outcome assessment. As limitations, there is the common issue of IHC regarding the selection of the representative tumoral block as there may be a significant variation between blocks (19, 31). We also did not measure VEGF expression in the tumoral specimens which might have been useful to correlate with the levels of CD105, as some studies report a parallel correlation (9, 11). Additionally, we were not able to analyze the differences of pre-existing tumor vessels after anti-angiogenic treatment due to the lack of available tumor tissue in the context of second recurrences. Furthermore, IDH status and MGMT methylation were not routinely performed at our center before 2016, and thus, this important prognostic information was not available (32, 33). Because of the retrospective nature of this study, there were some missing data that could have influenced our results.

## Conclusion

In this study, the higher expression of MVD-CD105 in recurrent GBM specimens seems to be associated with worse progression-free survival and overall survival in patients treated with anti-angiogenics upon recurrence, whereas CD105 expression in the primary tumor had no impact on survival outcomes. This highlights the importance of tracking the molecular evolution of the tumor over time. Further prospective studies are needed to confirm the prognostic value and the interest in the combined blockade of CD105 and VEGF in specific subgroups of patients.

## Data availability statement

The raw data supporting the conclusions of this article will be made available by the authors, without undue reservation.

## Ethics statement

The studies involving human participants were reviewed and approved by Local Ethical Committee of Centro Hospitalar 195 Universitaírio S. Joaão (Porto, Portugal) (no. 17/21). Written informed consent for participation was not required for this study in accordance with the national legislation and the institutional requirements.

## Author contributions

Conceptualization and design of the work: BC and JL. Acquisition and analysis of data: AB, BC, DL, and RS. Interpretation of data: AB, BC, RS, PL, RV, and JL. Original draft preparation: AB and BC. Review and editing and final approval of the manuscript: all authors.

## Conflict of interest

The authors declare that the research was conducted in the absence of any commercial or financial relationships that could be construed as a potential conflict of interest.

## Publisher’s note

All claims expressed in this article are solely those of the authors and do not necessarily represent those of their affiliated organizations, or those of the publisher, the editors and the reviewers. Any product that may be evaluated in this article, or claim that may be made by its manufacturer, is not guaranteed or endorsed by the publisher.
